# Dual oxidase 1 and NADPH oxidase 2 exert favorable effects in cervical cancer patients by activating immune response

**DOI:** 10.1186/s12885-019-6202-3

**Published:** 2019-11-09

**Authors:** Sang Yeon Cho, Sungha Kim, Mi-Ju Son, Gwanghun Kim, Parul Singh, Ha Neul Kim, Hei-Gwon Choi, Heon Jong Yoo, Young Bok Ko, Byung Seok Lee, Hyuk Soo Eun

**Affiliations:** 1 School of Medicine, Chungnam National University, 266 Munwha-ro, Jung-gu, Daejeon, Republic of Korea; 2 Department of Clinical Medicine, Korea Institute of Oriental Medicine, 1672 Yuseong-daero, Yuseong-gu, Daejeon, Republic of Korea; 3 Department of Anatomy, College of Medicine, Seoul National University, 103, Daehak-ro, Jongno-gu, Seoul, Republic of Korea; 4 Department of Microbiology and Immunology, School of Medicine, Chonbuk National University, 20 Geonji-ro, Jeonju, Republic of Korea; 5 Brain Korea 21 PLUS project for Medical Science, Chungnam National University, 266 Munwha-ro, Jung-gu, Daejeon, Republic of Korea; 6 Research Institute of Medical Sciences, School of Medicine, Chungnam National University, 266 Munwha-ro, Jung-gu, Daejeon, Republic of Korea; 7 Department of Obstetrics and Gynecology, Chungnam National University Hospital, 282 Munwha-ro, Jung-gu, Daejeon, Republic of Korea; 8 Department of Obstetrics and Gynecology, School of Medicine, Chungnam National University, 266 Munwha-ro, Jung-gu, Daejeon, Republic of Korea; 9 Department of Internal Medicine, Chungnam National University Hospital, 282 Munwha-ro, Jung-gu, Daejeon, Republic of Korea; 10 Department of Internal Medicine, School of Medicine, Chungnam National University, 266 Munwha-ro, Jung-gu, Daejeon, Republic of Korea

**Keywords:** NADPH oxidases, Dual oxidases, Uterine cervical neoplasms, Papillomaviridae, Survival, Disease-free survival

## Abstract

**Background:**

Nicotinamide adenine dinucleotide phosphate (NADPH) oxidase-derived reactive oxygen species (ROS) not only can promote cancer progression, but also they have recently emerged as mediators of the mucosal immune system. However, the roles and clinical relevance of the collective or individual NADPH oxidase (NOX) family genes in cervical cancer have not been studied.

**Methods:**

We investigated the clinical significance of the *NOX* family genes using data from 307 patients with cervical cancer obtained from The Cancer Genome Atlas. Bioinformatics and experimental analyses were performed to examine NOX family genes in cervical cancer patients.

**Results:**

*Dual Oxidase1 (DUOX1)* and *Dual Oxidase 2 (DUOX2)* mRNA levels were upregulated 57.9- and 67.5-fold, respectively, in cervical cancer patients. The protein expression of DUOX1, DUOX2, and NOX2 also identified in cervical squamous cell carcinoma tissues. Especially, *DUOX1* and *DUOX2* mRNA levels were significantly increased in patients infected with human papillomavirus (HPV) 16. Moreover, high *DUOX1* mRNA levels were significantly associated with both favorable overall survival and disease-free survival in cervical cancer patients. High *NOX2* mRNA levels was significantly associated with favorable overall survival. Gene set enrichment analyses revealed that high *DUOX1* and *NOX2* expression was significantly correlated with the enrichment of immune pathways related to interferon (IFN)-alpha, IFN-gamma, and natural killer (NK) cell signaling. Cell-type identification by estimating relative subsets of known RNA transcript analyses indicated that the fraction of innate immune cells, including NK cells, monocytes, dendritic cells, and mast cells, was elevated in patients with high *DUOX1* expression.

**Conclusions:**

*DUOX1* and *NOX2* expression are associated with mucosal immunity activated in cervical squamous cell carcinoma and predicts a favorable prognosis in cervical cancer patients.

## Background

Human papillomavirus (HPV) is the primary etiologic agent of cervical cancer [1]. However, HPV alone is not sufficient for tumor progression; the clinical manifestation of HPV infection depends on the immune response of the host [2]. Tumors are recognized by the immune system and their development can be stopped or controlled through a process known as immunosurveillance [3]. The mucosal epithelium represents the first line of defense against virus invasion. An immature or weakened innate immunity of the uterine cervical epithelium may exacerbate viral infection. Therefore, despite the improvements in vaccines against HPV, more studies are needed to identify new therapeutic inducers for the reinforcement of the innate immune responses against HPV infection in cervical cancer patients.

The NADPH oxidase (NOX) family, the major family of enzymes that catalyze reactive oxygen species (ROS) production, comprises seven members: NOX1–5, dual oxidase (DUOX) 1, and DUOX2 [4]. ROS induce oxidative stress and diverse inflammatory responses [5]. Excessive ROS production by NOX homologs as a result of chronic inflammation can also promote proliferative and invasive malignancies [6]. However, oxidative innate immune defense mechanism mediated by NADPH oxidase family members has been emerged, especially, DUOX plays an important role in host mucosal immunity by producing hydrogen peroxide [7–9]. Host-defense properties of DUOX have also been identified in non-mammalian organisms [10–13]. Homologs of DUOX are found in nearly all multicellular organisms, and DUOX enzymes seem to be evolved to fundamentally serve host immune defense [14]. DUOX1 and DUOX2 may have unique roles in specific arms of the innate immune response. Nevertheless, the immunologic effect of DUOX in the uterine cervical mucosa, which provides the first line of defense to HPV invasion, especially in cervical cancer, has not yet been investigated.

The present study aimed to investigate whether NOX family members are involved in cervical cancer progression or host immunity in response to cervical cancer. We used data from 307 cervical cancer patients obtained from The Cancer Genome Atlas (TCGA). Indeed, we discovered a prognostic value of *DUOX1* and *NOX2* expression in cervical cancer patients, and we attempted to elucidate the underlying mechanisms by using bioinformatics analyses, including gene set enrichment analysis (GSEA) and cell-type identification by estimating relative subsets of known RNA transcript (CIBERSORT). Moreover, we analyzed the protein expression of NOX2, DUOX1, and DUOX2 using clinical tissue samples from cervical cancer patients.

## Methods

### Gene and protein expression profiles

RNAseqV2-RSEM_genes and clinical data from 307 Cervical Squamous Cell Carcinoma and Endocervical Adenocarcinoma (CESC) samples and 3 normal control samples were obtained from The Cancer Genome Atlas (http://portal.gdc.cancer.gov/) and Firebrowse (http://firebrowse.org/) for gene expression analysis. The validation set (GSE75132) of 30 samples with persistent HPV 16 infection and 11 normal control samples was downloaded from the Gene Expression Omnibus (GEO) database (https://www.ncbi.nlm.nih.gov/geo/). Raw data were initially processed in R v.3.2.5 (http://www.r-project.org). Chip data were normalized with RankNormalize in GenePattern (http://www.broadinstitute.org/cancer/software/genepattern/). Gene Expression Profiling Interactive Analysis (GEPIA; http://gepia.cancer-pku.cn/) was utilized to compare mRNA expression between cervical cancer patients based on data from TCGA database (https://portal.gdc.cancer.gov/) and 13 normal controls based on data from The Genotype-Tissue Expression (GTEx) Project from the Broad Institute of MIT and Harvard (www.gtexportal.org). Human normal tissue distribution of *DUOX1*, *DUOX2*, and *NOX2* was analyzed based on RNAseq data extracted from the GTEx project. Protein expression and immunohistochemical (IHC) staining data were obtained from the Human Protein Atlas (HPA) (http://www.proteinatlas.org).

### Western blotting

Total protein samples were isolated from frozen liver tissue using RIPA lysis buffer, containing protease and phosphatase inhibitor cocktail (TransLab, #30-04CLI19SSH). Samples were separated in a 10% SDS-polyacrylamide gel electrophoresis and transferred onto nitrocellulose membrane (GE Healthcare Life Sciences, #10600023). After the membranes were blocked in 5% skim milk for 1 h at room temperature, they were incubated with primary antibodies overnight at 4 °C and then with the corresponding secondary antibodies for 1 h at room temperature. All of the primary antibodies gp91-phox antibody (Santa Cruz Biotechnology, #K0817) and β-actin (Cell Signaling, #4970 s) were used at a dilution of 1:1000 except DUOX1 (Santa Cruz Biotechnology, #B2817) (1:500) and DUOX2 (Santa Cruz Biotechnology, #D0317) (1:500). Secondary antibodies were used at 1:2500 dilution. Immunoreactive bands were detected using the enhanced chemiluminescence (ECL) detection system with a PhosphorImager (GE Healthcare). Protein expression levels were normalized to the levels of the β-actin, which was used as a loading control.

### Patients samples

Frozen cervical cancer tissue samples were obtained from some of patients with cervical cancer and their controls were obtained from the cohort of the Department of Obstetrics and Gynecology, Chungnam National University Hospital (Daejeon, South Korea) and were analyzed by western blot. In this study, each three normal cervical cancer tissues, early-stage cervical squamous cell carcinoma, advanced-stage cervical squamous cell carcinoma, and endocervical adenocarcinoma tissues deposited with the Human Resources Bank of Korea in Chungnam National University Hospital were used for this study. Authorization for the use of these tissues for research purposes and ethical approval were obtained from the Institutional Review Board of Chungnam National University Hospital (IRB number: 2019-05-087). Written informed consents, which were approved by Institutional Review Board of Chungnam National University Hospital, were received from the entire patients who had provided the tissue.

### Functional enrichment analysis

Gene Set Enrichment Analysis (GSEA) was used to assess enrichment of mRNAs associated with Hallmark and Kyoto Encyclopedia of Genes and Genomes (KEGG) pathways sets [15]. GSEA was conducted using the 10% of CESC samples with the most strongly upregulated *DUOX1* and *NOX2* expression and the 10% of samples with the most strongly downregulated *DUOX1* and *NOX2* expression. Enrichment maps were visualized in Cytoscape v.3.5.1 (www.cytoscape.org). A *p*-value of less than 0.05 was considered significant.

### Analysis of immune cell subsets from mRNA expression profiles

To quantify the relative abundances of 22 tumor-associated leukocyte subsets in samples from HPV-positive and -negative CESC patients, we utilized the Cell type Identification By Estimating Relative Subsets Of known RNA Transcript (CIBERSORT) method and the LM22 gene signature, which allow for highly sensitive and specific discrimination of hematopoietic cells and were well-designed and validated based on gene expression profiles from Affymetrix Human Genome U133A/Plus2 [16]. CIBERSORT analysis was conducted using the 10% samples with the most strongly upregulated *DUOX1* and *NOX2* expression and the 10% of samples with the most strongly downregulated *DUOX1* and *NOX2* expression.

### Survival analysis

Survival analysis of cervical cancer patients was performed using GEPIA. The cumulative event (death) rate was calculated by the Kaplan–Meier method, using the time from the date of operation to the date of death as the outcome variable. Survival curves stratified by risk factors were compared by log-rank test, with *p*-values less than 0.05 considered to indicate statistical significance. The median group cutoff was median.

### Statistical analysis

Data were analyzed in Prism version 5.0 (GraphPad Prism Software, La Jolla, CA, USA) and Statistical Package for Social Sciences for Windows version 13.0 (SPSS, Chicago, IL, USA). Distributions between two groups were compared by *t*-test (or by Kolmogorov-Smirnov test when the expected frequency in any group was less than 5) for continuous variables, and by Chi-square test (or Fisher’s exact test when the expected frequency in any group was less than 5) for categorical variables. Three or more groups were compared by one-way analysis of variance. A *p*-value of less than 0.05 was considered significant.

## Results

### *DUOX1* and *DUOX2* are predominantly expressed in cervical cancer patients

**Fig. 1:**
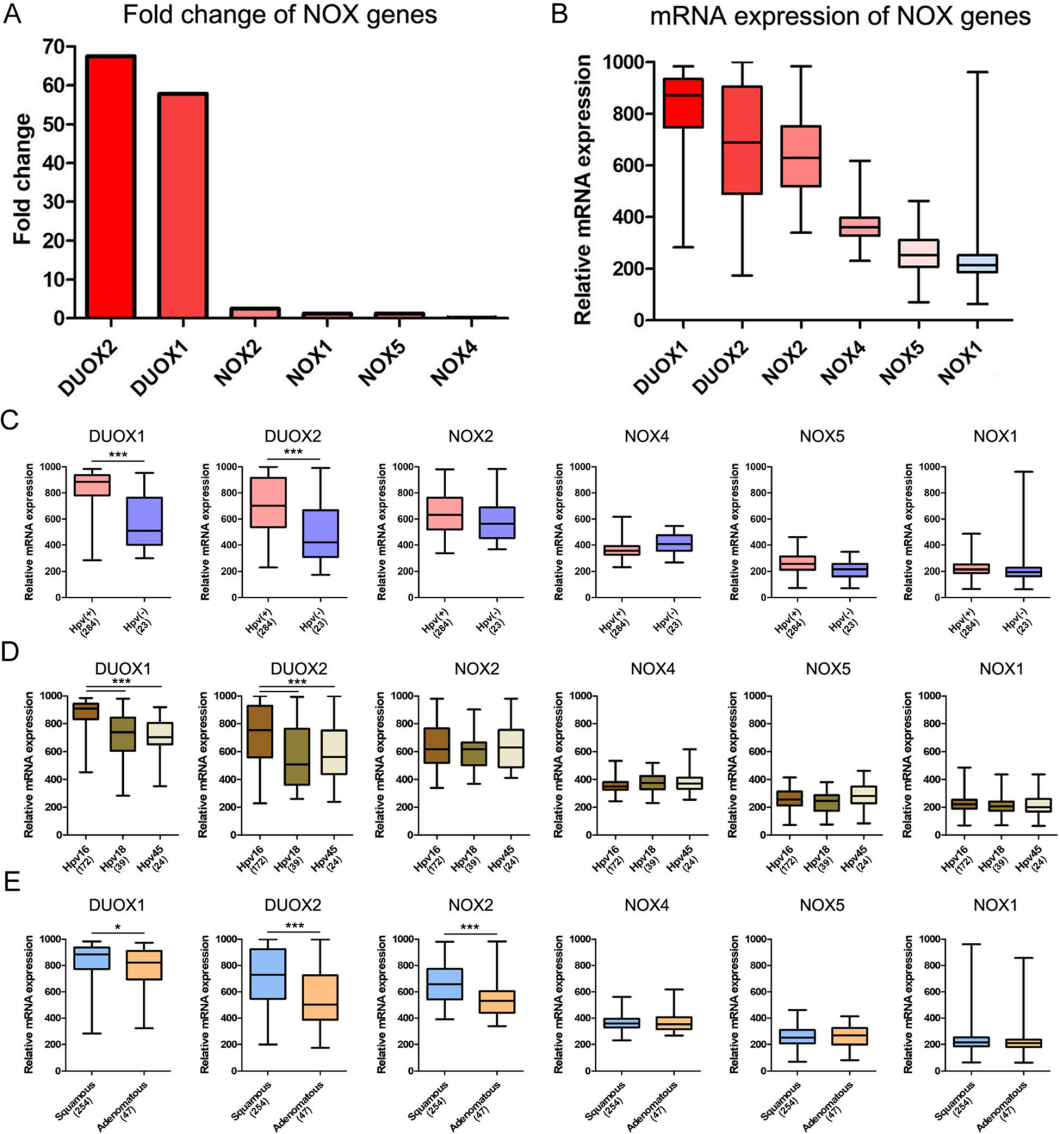
mRNA and protein expression of *NOX* genes in cervical cancer. **a** Fold change in mRNA expression in comparison to normal control levels. Data are from The Cancer Genome Atlas. **b** Relative mRNA expression of *NOX* genes in cervical cancer patients. **c** mRNA expression in patients with and without HPV infection. **d** mRNA expression among patients with HPV 16, HPV 18, and HPV 45. **e** mRNA expression according to histologic type in squamous cell carcinoma and adenocarcinoma. Six adenosquamous carcinoma cases were excluded from histologic comparison. *p < 0.05; **p < 0.01; ***p < 0.001 by one-way ANOVA to compare more than two groups, or by *t*-test to compare two groups

Clinicopathological characteristics of the patients are listed in Table 1. mRNA and protein expression of DUOX and NOX genes was examined in patients with cervical cancer (Fig. 1). *DUOX1* and *DUOX2* expression was increased by 57.9- and 67.5-fold, respectively, whereas *NOX4* expression was decreased by 0.17-fold in patients compared to normal control subjects (Fig. 1a). DUOX1, DUOX2, and NOX2 protein expression were also identified in our clinical cervical cancer samples (Additional file 1). *DUOX1* and *DUOX2* were also the most abundant *NOX* transcripts in cervical cancer patients, whereas *NOX3* was the least abundant and was undetectable in normal control subjects (Fig. 1b and Table 2). *DUOX* and *NOX* mRNA expression was significantly different according to the presence of HPV infection and histologic type. In cervical cancer patients with HPV infection, *DUOX1* and *DUOX2* mRNA levels were significantly increased as compared to patients without HPV infection (Fig. 1c). *DUOX1* and *DUOX2* mRNA levels were significantly higher in patients with HPV 16 than in patients with HPV 18 and HPV 45 (Fig. 1d). In addition, mRNA and protein levels of DUOX1 and DUOX2 were higher in patients with cervical squamous cell carcinoma than in those with endocervical adenocarcinoma (Fig. 1e and Additional file 1). However, mRNA levels of NOX family members were not significantly associated with clinical stage and pathologic stage (Additional file 2). Moreover, mRNA expression of *DUOX1*, *DUOX2*, and *NOX2* was also significantly increased according to the GEPIA database, as shown in Additional file 3. The normal tissues distribution of human DUOX1, DUOX2, and NOX2 is illustrated in Additional file 4.

**Table 1:** Clinicopathologic information of the cervical cancer patients

Feature	Total (%)
Number	307 (100)
Age	
≤ 50 years	188 (61.2)
> 50 years	119 (38.8)
Histological type	
Squamous cell carcinoma	254 (82.7)
Endocervical adenocarcinoma	47 (15.3)
Adenosquamous carcinoma	6 (2.0)
Vital status	
Alive	235 (76.5)
Dead	72 (23.5)
Postoperative Treatment	
Yes	103 (33.6)
No	77 (25.1)
Clinical stage	
I	163 (53.1)
II	70 (22.8)
III	46 (15.0)
IV	21 (6.8)
Morphological type	
Non-keratininzing type	120 (39.1)
Keratininzing type	55 (17.9)
Lymphatic invasion	
Absent	72 (23.5)
Present	80 26.1)
Human papilloma virus status	
Negative	23 (7.5)
Positive (High risk)	284 (92.5)
Hpv 16	172 (56.0)
Hpv 18	39 (12.7)
Hpv 45	24 (7.8)
Hpv etc	47 (15.3)

**Table 2:** Expression of the NADPH oxidase family in patients with cervical cancer

Symbol	Gene name	Aliases	Chromosome location	Log fold change
NOX1	NADPH Oxidase 1	Mitogenic Oxidase (Pyridine Nucleotide-Dependent Superoxide-Generating)	Xq22.1	1.21
NOX2	NADPH Oxidase 2	CYBB (Cytochrome B-245 Beta Chain), Superoxide-Generating NADPH Oxidase Heavy Chain Subunit, Heme-Binding Membrane Glycoprotein Gp91phox, Neutrophil Cytochrome B 91 KDa Polypeptide	Xp21.1	2.50
NOX3	NADPH Oxidase 3	Mitogenic Oxidase 2, NADPH Oxidase Catalytic Subunit-Like 3	6q25.3	NA
NOX4	NADPH Oxidase 4	Kidney Superoxide-Producing NADPH Oxidase, Kidney Oxidase-1	11q14.3	0.17
NOX5	NADPH Oxidase 5	NADPH Oxidase, EF-Hand Calcium Binding Domain 5	15q23	1.21
DUOX1	Dual Oxidase 1	NADPH Thyroid Oxidase 1, Nicotinamide Adenine Dinucleotide Phosphate Oxidase, Flavoprotein NADPH Oxidase, Large NOX 1, Long NOX 1	15q21.1	57.9
DUOX2	Dual Oxidase 2	NADPH Thyroid Oxidase 2, Nicotinamide Adenine Dinucleotide Phosphate Oxidase	15q21.1	67.5

### Cervical cancer patients with high expression of *DUOX1* and *NOX2* have a favorable prognosis

**Fig. 2:**
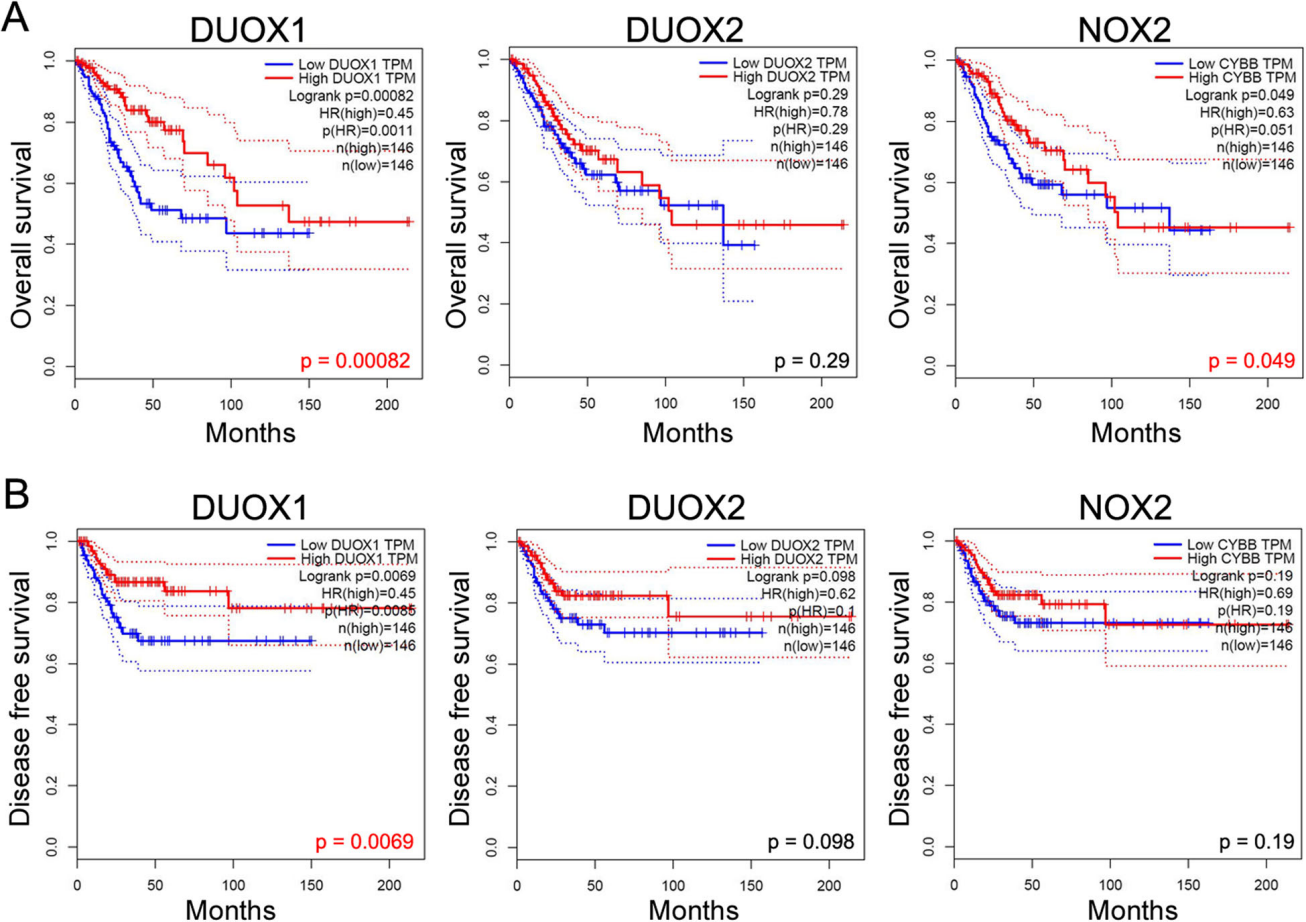
Survival analysis of cervical cancer patients based on GEPIA data. **a** Kaplan–Meier survival analysis conducted with high and low mRNA expression of *DUOX1*, *DUOX2*, and *NOX2* regarding their associations with overall survival (**b**) Kaplan–Meier survival analysis conducted with high and low mRNA expression of *DUOX1*, *DUOX2*, and *NOX2* regarding their associations with disease-free survival

Based on the log-rank test in GEPIA, abundant mRNA expression of *DUOX1* (hazard ratio 0.45, 95% confidence interval, *p =* 0.00082) and *NOX2* (hazard ratio 0.63, 95% confidence interval, *p* = 0.049) was significantly associated with better prognosis of CESC patients in terms of overall survival (Fig. 2a). High mRNA expression of *DUOX1* (hazard ratio 0.45, 95% confidence interval, *p* = 0.0069) was significantly associated with better prognosis of CESC patients in disease-free survival (Fig. 2b). In addition, *NOX1*, *NOX4*, and *NOX5* mRNA levels were not significantly associated with the prognosis of cervical cancer patients.

### Immune pathways strongly associated with *DUOX1* and *NOX2* expression

**Fig. 3:**
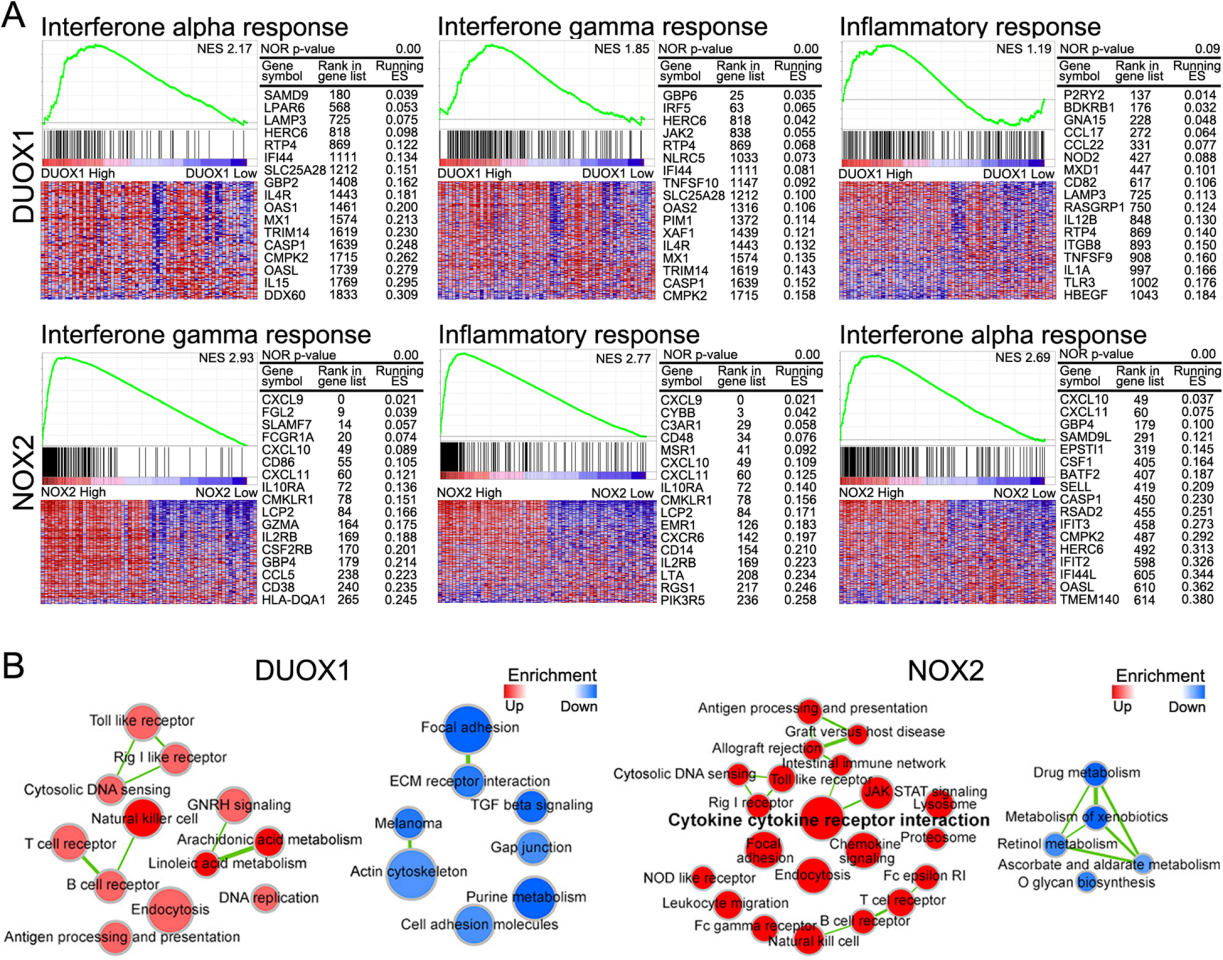
Gene set enrichment analysis and map visualization for *DUOX1* and *NOX2* in cervical cancer. **a** Representative GSEA data with *p* values for *DUOX1* and *NOX2* was shown. **b** Enrichment maps of *DUOX1* and *NOX2* in KEGG pathways. Red nodes represent enrichment in the former, whereas blue nodes represent enrichment in the latter. Color intensity is proportional to the degree of enrichment, and clusters represent functionally related gene sets. Data are for the 10% of samples with the most strongly upregulated *DUOX1* and *NOX* expression and the 10% of samples with the most strongly downregulated *DUOX1* and *NOX* expression. The NES (Normalized Enrichment Score) computes the density of modified genes in the dataset with the random expectancies, normalized by the number of genes found in a given gene cluster, to take into account the size of the cluster

Using GSEA and enrichment network visualization, enrichment of mRNAs associated with Hallmark pathways and KEGG pathways (Fig. 3) were investigated in the 10% CESC samples with the most upregulated *DUOX1* and *NOX2* expression and in the 10% of samples with the most downregulated *DUOX1* and *NOX2* expression. In Hallmarks pathways, high *DUOX1* and *NOX2* mRNA expression was significantly associated with immune pathways related to interferon (IFN)-alpha and IFN-gamma (Fig. 3a and Table 3). The NES (Normalized Enrichment Score) values of IFN-alpha and -gamma responses associated with *DUOX1* were 2,17 and 1.85. The NES values of IFN-gamma, inflammatory response, and IFN-alpha responses related with *DUOX2* were 2,93, 2.77, and 2.69, respectively.

**Table 3:** Hallmark pathways of DUOX1 and NOX2 in cervical cancer

Term	Size	ES	NES	NOM *p*-val
DUOX1 – Hallmark pathways up				
HALLMARK_INTERFERON_ALPHA_RESPONSE	97	0.57	2.17	0.00
HALLMARK_INTERFERON_GAMMA_RESPONSE	199	0.44	1.85	0.00
HALLMARK_ESTROGEN_RESPONSE_EARLY	197	0.39	1.63	0.00
HALLMARK_ESTROGEN_RESPONSE_LATE	200	0.37	1.56	0.00
HALLMARK_INFLAMMATORY_RESPONSE	200	0.29	1.19	0.09
HALLMARK_TNFA_SIGNALING_VIA_NFKB	200	0.27	1.12	0.17
DUOX1 – Hallmark pathways down				
HALLMARK_EPITHELIAL_MESENCHYMAL_TRANSITION	199	−0.59	−2.43	0.00
HALLMARK_ANGIOGENESIS	36	−0.51	−1.62	0.01
HALLMARK_HEDGEHOG_SIGNALING	36	−0.47	−1.49	0.04
HALLMARK_KRAS_SIGNALING_UP	200	−0.34	−1.41	0.01
HALLMARK_WNT_BETA_CATENIN_SIGNALING	42	−0.39	− 1.29	0.11
HALLMARK_APICAL_JUNCTION	200	−0.30	−1.27	0.03
NOX2– Hallmark pathways up				
HALLMARK_INTERFERON_GAMMA_RESPONSE	199	0.80	2.93	0.00
HALLMARK_INFLAMMATORY_RESPONSE	200	0.77	2.77	0.00
HALLMARK_INTERFERON_ALPHA_RESPONSE	97	0.81	2.69	0.00
HALLMARK_IL6_JAK_STAT3_SIGNALING	87	0.76	2.47	0.00
HALLMARK_IL2_STAT5_SIGNALING	198	0.63	2.30	0.00
HALLMARK_TNFA_SIGNALING_VIA_NFKB	200	0.63	2.29	0.00
NOX2– Hallmark pathways down				
HALLMARK_GLYCOLYSIS	199	−0.36	−1.50	0.00
HALLMARK_NOTCH_SIGNALING	32	−0.34	−1.05	0.37
HALLMARK_HEDGEHOG_SIGNALING	36	−0.28	−0.89	0.65
HALLMARK_FATTY_ACID_METABOLISM	156	−0.22	−0.88	0.80
HALLMARK_PROTEIN_SECRETION	96	−0.22	−0.83	0.89
HALLMARK_G2M_CHECKPOINT	194	−0.12	−0.52	1.00

In KEGG pathways, genes associated with immune pathways, including NK cells, T-cell receptor, B-cell receptor, cytosolic DNA sensing, Toll-like receptor, and retinoic acid-inducible gene-I (RIG-I) receptor were significantly enriched under high *DUOX1* mRNA expression*.* However, repression of *DUOX1* mRNA expression significantly enriched for genes related with cancer-related pathways, including focal adhesion, extracellular matrix receptor interaction, transforming growth factor-beta signaling, and cell adhesion (Fig. 3b). Meanwhile, *NOX2* expression enriched for several immune pathways associated with cytokine cytokine-receptor interactions, Janus kinase/signal transducers and activators of transcription (JAK/STAT) signaling, intestinal immunity, Toll-like receptor signaling, RIG-I receptor signaling, cytosolic DNA sensing, T cell receptor, B cell receptor, and NK cell signaling. However, drug-, xenobiotic-, and retinol-metabolic pathways were significantly enriched in samples with downregulated *NOX2* mRNA expression (Fig. 3b).

### Innate and adaptive immune cell subsets are increased in patients with high *DUOX1* and *NOX2* expression

**Fig. 4:**
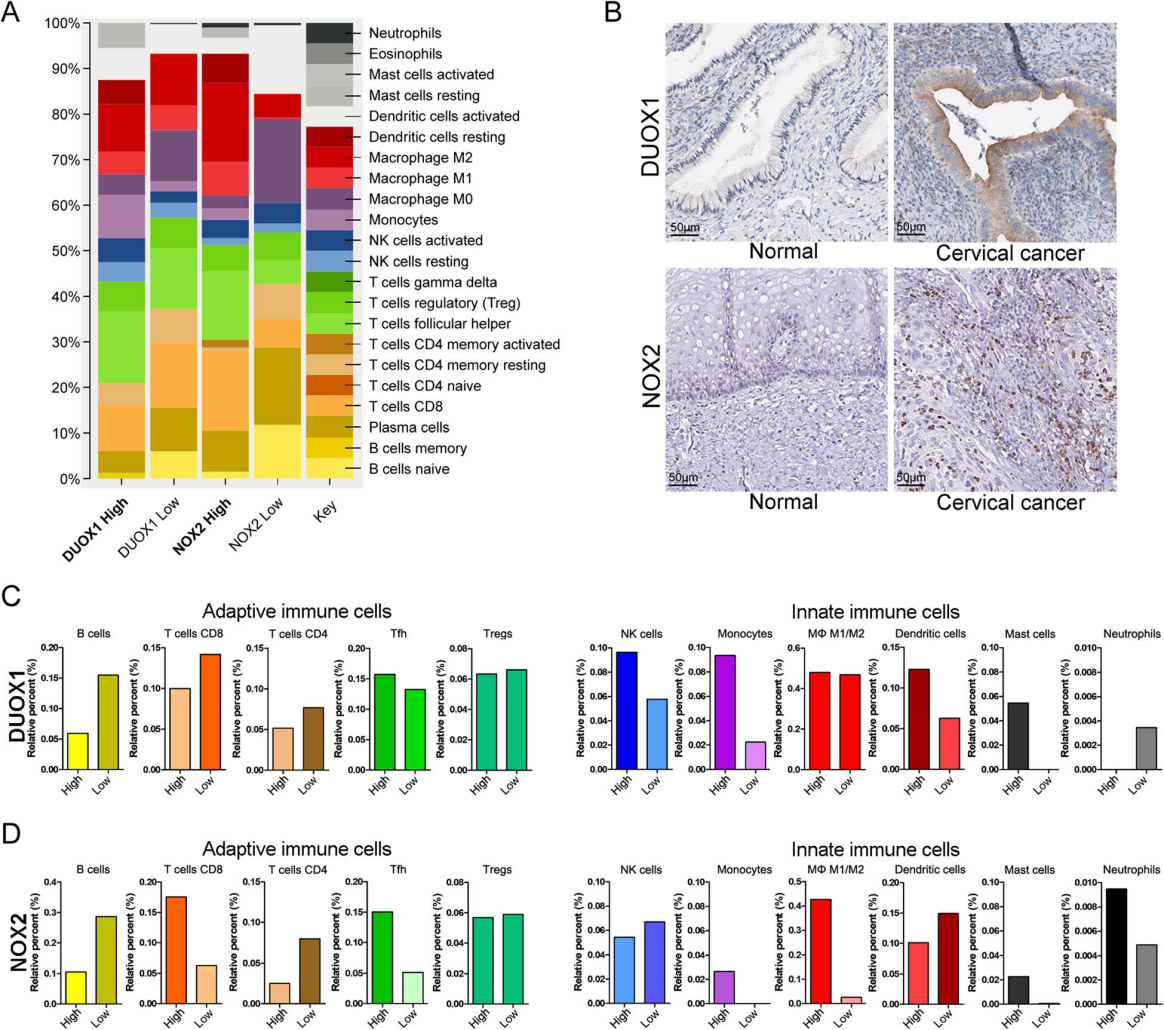
Immune cell signatures in cervical cancer patients with *DUOX1* and *NOX2* expression. Estimated mRNA percentages of 22 immune cell subsets (LM22 signature), as calculated by CIBERSORT, in cervical cancer patients with *DUOX1* and *NOX2* gene expression. **a** Relative percentages of LM 22 signature subsets in patients with *DUOX1* and *NOX2* gene expression. **b** Immunohistochemical staining of DUOX1 and NOX2 adapted from Human Protein Atlas. **c** Relative percentages of immune cells in patients with high and low *DUOX1* mRNA expression. **d** Relative percentages of immune cells in patients with high and low *NOX2* mRNA expression

CIBERSORT was used to estimate the abundances of immune cell subsets and evaluate the changes in immune cell subsets within tumor micro-environment in cervical cancer (Fig. 4 and Additional files 5 and 6). The analysis was carried out using the 10% samples with the highest and lowest *DUOX1* and *NOX2* expression, and revealed a change in abundance in 22 immune cell types (Fig. 4a). Furthermore, the IHC staining of DUOX1 and NOX2 protein was examined in cervical cancer based on data from the Human Protein Atlas (Fig. 4b). It is discovered that the IHC staining of DUOX1 was increased in secretary cells of uterine cervical glands in cervical cancer tissues. The NOX2 was selectively stained intraepithelial infiltrating cells in cervical cancer tissue (Fig. 4b).

Next, we specifically investigated the changes in abundance of adaptive and innate immune cells. Increased abundances of innate immune cells, including NK cells, monocytes, dendritic cells, and mast cells, and decreased abundances of adaptive immune cells, including B cells, CD8 T cells, and CD4 T cells, were identified in the patients with high *DUOX1* expression compared to the patients with low *DUOX1* expression (Fig. 4c). Additionally, in the validation data set, high mRNA levels of *DUOX1* were also associated with increased innate immune cells, including NK cells and mast cells, and a decreased fraction of B cells (Additional file 5). On the other hand, increased percentages of CD8 T cells and follicular helper T cells and decreased percentages of B cells and CD4 T cells in adaptive immune cells were identified in patients with *NOX2* high expression (Fig. 4d and Additional file 6). In innate immune cells, the M1/M2 macrophage ratio and neutrophils were increased in patients with high *NOX2* expression (Additional file 6).

## Discussion

We tried to identify new therapeutic targets for the reinforcement of immune responses against HPV infection. This study was the first to examine the immunologic role and clinical significance of NADPH oxidase family members in cervical cancer patients. We initially evaluated *DUOX1* and *DUOX2* mRNA levels in the normal ectocervix, endocervix, and vagina (Additional file 4). Interestingly, we found that *DUOX1* and *DUOX2* mRNA levels were dramatically increased in cervical cancer patients infected with HPV 16 (Fig. 1d). DUOX1 and DUOX2 protein expression were also identified in cervical squamous cell carcinoma (Additional file 1). In line with our findings, a previous study reported that *DUOX* and *DUOX*-derived ROS were upregulated in the respiratory mucosa upon influenza virus infection [17]. Moreover, in our study, high expression levels of *DUOX1* mRNA were significantly associated with favorable overall survival as well as disease-free survival in cervical cancer patients. Indeed, several studies were reported that the relationship between expression and prognostic effect of DUOX1 depend on the cancer tissue type. For example, DUOX enzymes were first identified in thyroid tissues and were found to be involved in thyroid hormone biosynthesis [18–20]. In thyroid cancer, DUOX1 is upregulated upon radiation, and DUOX1-dependent H_2_O_2_ production promotes persistent DNA damage and genome instability, which might contribute to cancer development [21, 22]. In contrast, in the respiratory tract, DUOX1 is mostly expressed in the tracheal and bronchial epithelium [9], and DUOX1 mRNA and protein are suppressed in lung cancer as a consequence of hypermethylation in the promoter region, and this suppression is associated with poor prognosis [23–25]. Moreover, *DUOX1* expression is low in the gastrointestinal tract and has been detected in the stomach lining [24, 26]. In gastric cancer, mRNA expression of *DUOX1* was downregulated, whereas, high levels of *DUOX1* mRNA were correlated with poor prognosis, paradoxically [27]. It is conceivable that the expression and prognostic effect of DUOX1 depend on the organ and cancer type.

The role of DUOX2 has been actively investigated in various malignancies [6, 23]. DUOX2 is the main isoform within the gastrointestinal tract and is expressed most prominently within the colon epithelium and rectal glands [9, 28]. It has been reported that strong DUOX2 expression accelerates the development of colorectal and pancreatic cancers in patients with inflammatory bowel disease and chronic pancreatitis, respectively [6]. Overexpression of DUOX2/DUOX2A during ulcerative colitis is also thought to be responsible for oxidative DNA damage, which predisposes these patients to colon cancer development [29]. However, in our study, *DUOX2* mRNA was detected in the vagina, and rarely detected on the cervix (Additional file 4). DUOX2 mRNA was also dramatically increased in cervical cancer patients; however, high *DUOX2* mRNA level was not associated with significant favorable prognosis. Moreover, *NOX2* mRNA was rarely detected on the cervix and vagina (Additional file 4). However, *NOX2* mRNA was significantly increased in cervical cancer patients with HPV, and high *NOX2* mRNA level was significantly associated with favorable overall survival. NOX2 protein expression were also identified in cervical squamous cell carcinoma and adenocarcinoma (Additional file 1). Indeed, it has been indicated that high levels of *NOX2* mRNA are implicated in promoting oncogenic characteristics in breast cancer, rectal cancer, and prostate cancer [30–32].

We conducted GSEA to verify the effects of *DUOX1* and *NOX2* on survival in cervical cancer patients. Notably, expression of both *DUOX1* and *NOX2* was significantly associated with immune pathways related to IFN-alpha and IFN-gamma. IFN is well known to be important for tumor suppression because it not only directly kills tumor cells, but also activates immune cells in the tumor microenvironment [33]. In addition, estrogen response and NK cell signaling pathways were closely related to *DUOX1* expression. Moreover, the pathways of TNF alpha and cytokine–cytokine receptor interaction were closely related to *NOX2* expression (Table 3). The effects of *DUOX1* and *NOX2* on survival in cervical cancer patients depend commonly on IFN-alpha and IFN-gamma, and differential pathways of *DUOX1* and *NOX2* were identified.

We investigated IHC staining of DUOX1 and NOX2 in cervical cancer tissues based on data from the Human Protein Atlas. Specifically, we discovered that DUOX1 and NOX2 staining in uterine cervical glands and intraepithelial infiltrating cells in cervical cancer tissues. These findings are supported by several recent reports on the presence of DUOX1 in non-epithelial cell types such as T-cells [34], macrophages [35], and innate lymphoid cells [36], and the presence of NOX2 in phagocytes [37]. To investigate the immune cell types regulated by *DUOX1* and *NOX2* mRNA expression in cervical cancer tissues more specifically, we utilized CIBERSORT analysis. Notably, high mRNA levels of *DUOX1* were closely related with increased innate immune cells, especially, NK cells, monocytes, dendritic cells, and mast cells, and also with a decreased fraction of adaptive immune cells, including B cells, CD8+ T, and CD4+ T cells. This indicates that *DUOX1* expression is highly associated with the innate immune cell response in cervical cancer. Recent evidence indicates that DUOX1 is expressed in innate lymphoid cells, where it has potential roles in innate lymphoid cell polarization, indicating broad host defense functions of DUOX1 [36]. Moreover, the patients with high mRNA expression levels of *NOX2* were closely related with increased fractions of M1/M2 macrophages and neutrophils among innate immune cells. In addition, the patients with high mRNA expression levels of *NOX2* mRNA levels were related with increased percentages of CD8+ T cells and follicular helper T cells among adaptive immune cells. These findings indicate that NOX2 expression is not only associated with phagocytes, such as macrophages and neutrophils [37], but also with adaptive immune cells, including CD8+ and follicular helper T cells. Based on GSEA and CIBERSORT analysis, it is suggested that *DUOX1* and *NOX2* have differential effects on the immune cell-mediated response in cervical cancer patients. In the tumor microenvironment, different types of infiltrating immune cells, including macrophages, dendritic cells, mast cells, NK cells, B cells, and effector T cells have diverse effects on cancer progression [38]. Especially, NK cells collaborate with dendritic cells to induce an immune response against viral infections and tumors [39]. Activated dendritic cells also play an important role in tumor therapy by acting as natural adjuvants, and tumor-specific follicular helper T cells act as potent antigen-presenting cells [40, 41]. In addition, an increased population of mast cells was related with favorable prognosis [42]. In this study, the increased mRNA levels of *DUOX1*, *DUOX2,* and *NOX2* in cervical cancer were identified in TCGA and GEO databases. Moreover, the protein expression and their localization of DUOX1 and NOX2 were also confirmed in our own patient samples and Human Protein Atlas database, respectively. However, analyses presented here are mainly suggested on the basis of different databases and there was still a challenge to experimentally validate the proposed underlying mechanism in a large cohort of cervical cancer patients.

## Conclusions

Our results suggest that DUOX1 and NOX2 mediate the IFN-based immune defense against HPV infection, and thereby affect the outcomes of cervical cancer patients. This study has extended our knowledge of the roles of DUOX1 and NOX2 in cervical cancer and shed light on its potential clinical use in cervical cancer patients. The approach of inducing a DUOX1 and NOX2-mediated immune response in uterine cervical mucosa is clinically expected to reinforce immune response to HPV infection and thus increase the survival of cervical cancer patients.

## Supplementary information

**Additional file 1.** Protein expression of DUOX1, DUOX2, and NOX2 in normal cervix tissues and cervical cancer tissues. (A) Protein expression in normal samples, squamous cell carcinoma and adenocarcinoma. (B) Clinicopathologic information for normal cervix patients and cervical cancer patients.

**Additional file 2. ***NOX* family members expression in clinical parameters. (A) mRNA expression in three clinical stage. (B) mRNA expression in pathologic stage (T for tumor size, N for nodal status, and M for status of tumor metastasis).

**Additional file 3. ***NOX* family members expression in CESC, based on GEPIA database (Gene Expression Profiling Interactive Analysis).

**Additional file 4. **Tissue distribution of *DUOX1*, *DUOX2*, and *NOX2* expression. RNAseq data were extracted from public data deposited by the Broad Institute of MIT and Harvard in the Gene Tissue Expression (GTEx) project.

**Additional file 5. **mRNA expression and Immune cell signatures in the validation data set (GSE75132). **(A)** mRNA expression of *DUOX2* and *NOX2* in patients with HPV 16 infection and normal control samples. (**B**) Relative percentages of LM 22 signature subsets in patients with *DUOX1* gene expression. **(C)** Relative percentages of immune cells in patients with high and low *DUOX1* gene expression. **(D)** Estimated percentage values of LM22 signature subsets, as calculated by CIBERSORT.

**Additional file 6. **Estimated percentage values of 22 immune cell signature (LM22 signature) subsets, as calculated by CIBERSORT, between CESC patient groups in cervical cancer patients with *DUOX1* and *NOX2* gene expression.

## Data Availability

The data set is available in Gene Expression Omnibus (GEO) (https://www.ncbi.nlm.nih.gov/geo/) with the accession number: GSE75132.

## Abbreviations

CESC: Cervical squamous cell carcinoma and endocervical adenocarcinoma
CIBERSORT: Cell type identification by estimating relative subsets of known RNA
DUOX1: Dual oxidase 1
DUOX2: Dual oxidase 2
ECL: Enhanced chemiluminescence
GEO: Gene Expression Omnibus
GEPIA: Gene Expression Profiling Interactive Analysis
GTEX: Genotype-Tissue Expression
HPA: Human Protein Atlas
IFN: Interferon
IHC: Immunohistochemical
JAK/STAT: Janus kinase/signal transducers and activators of transcription
KEGG: Kyoto Encyclopedia of Genes and Genomes
NADPH: Nicotinamide adenine dinucleotide phosphate
NES: Normalized Enrichment Score
NK: Natural killer
NOX: Nicotinamide adenine dinucleotide phosphate oxidase
RIG-I: Retinoic acid-inducible gene I
ROS: Reactive oxygen species
TCGA: The Cancer Genome Atlas
