# Supplementary figures and images for: Dual oxidase 1 and NADPH oxidase 2 exert favorable effects in cervical cancer patients by activating immune response
Source: BMC Cancer. 2019 Nov 9;19:1078. doi: 10.1186/s12885-019-6202-3 (PMC6842485; doi:10.1186/s12885-019-6202-3)

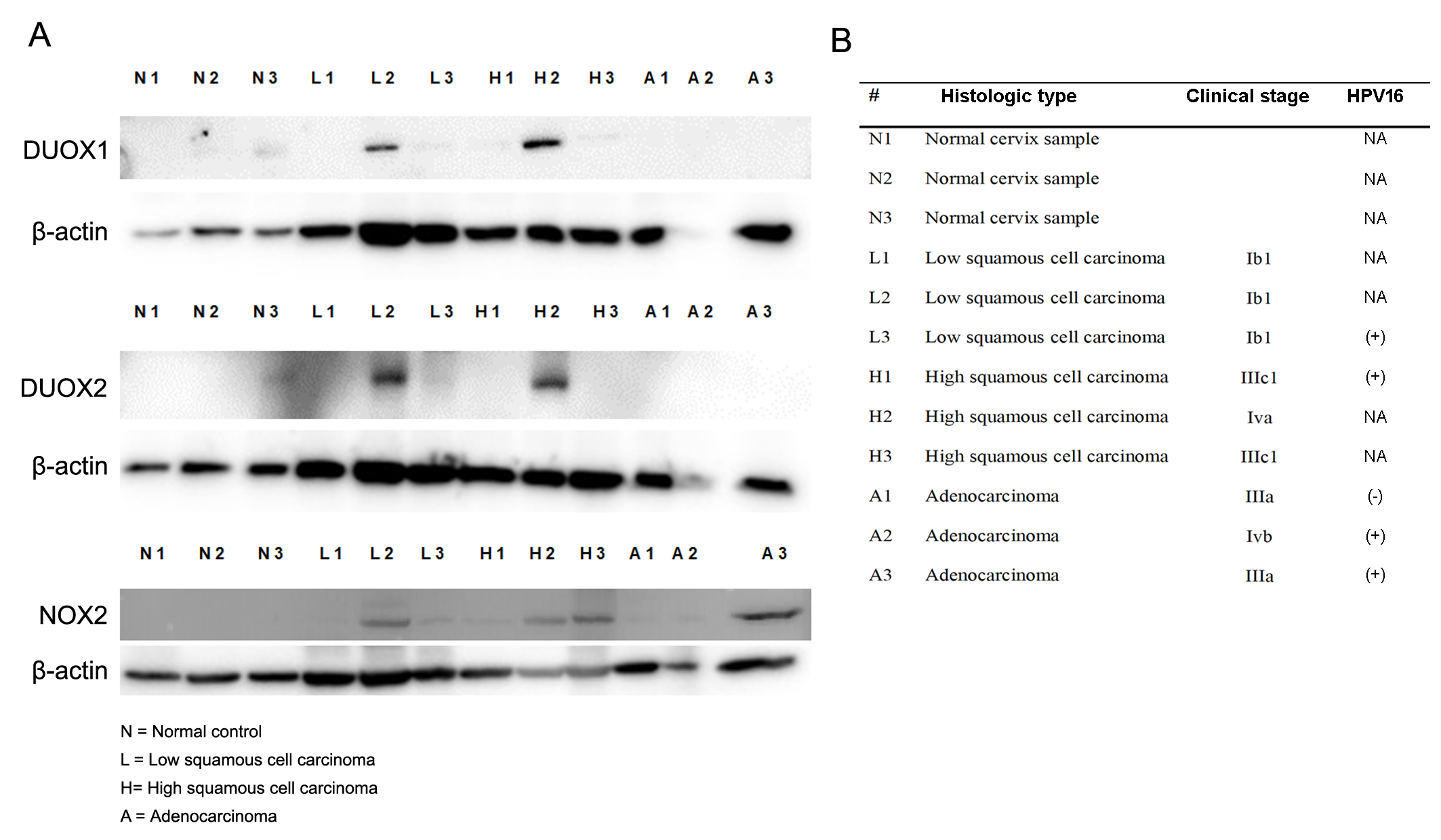

Supplement: Supplementary file 1 — Additional file 1. Protein expression of DUOX1, DUOX2, and NOX2 in normal cervix tissues and cervical cancer tissues. (A) Protein expression in normal samples, squamous cell carcinoma and adenocarcinoma. (B) Clinicopathologic information for normal cervix patients and cervical cancer patients. [file 12885_2019_6202_MOESM1_ESM.tif]

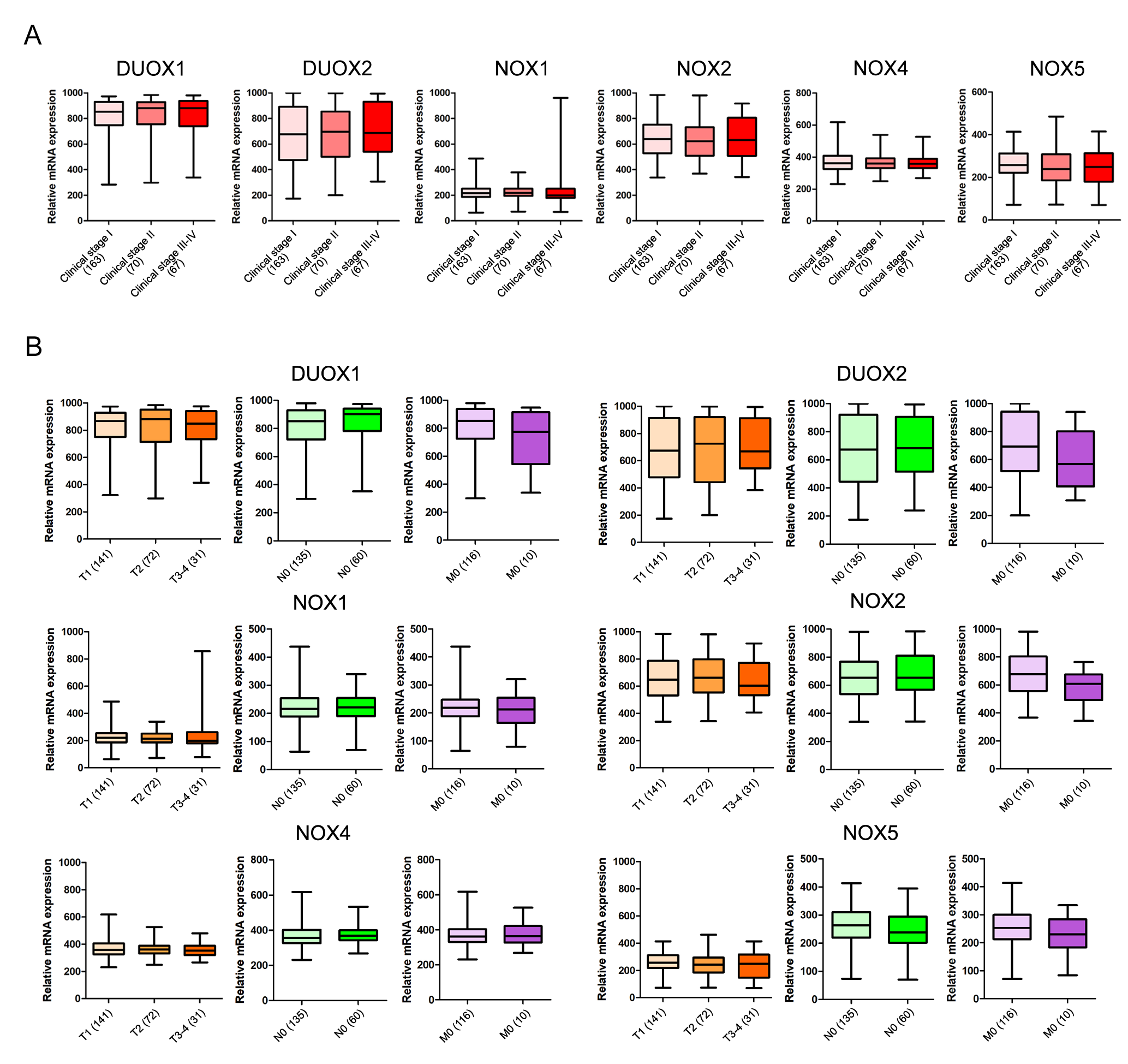

Supplement: Supplementary file 2 — Additional file 2. NOX family members expression in clinical parameters. (A) mRNA expression in three clinical stage. (B) mRNA expression in pathologic stage (T for tumor size, N for nodal status, and M for status of tumor metastasis). [file 12885_2019_6202_MOESM2_ESM.tif]

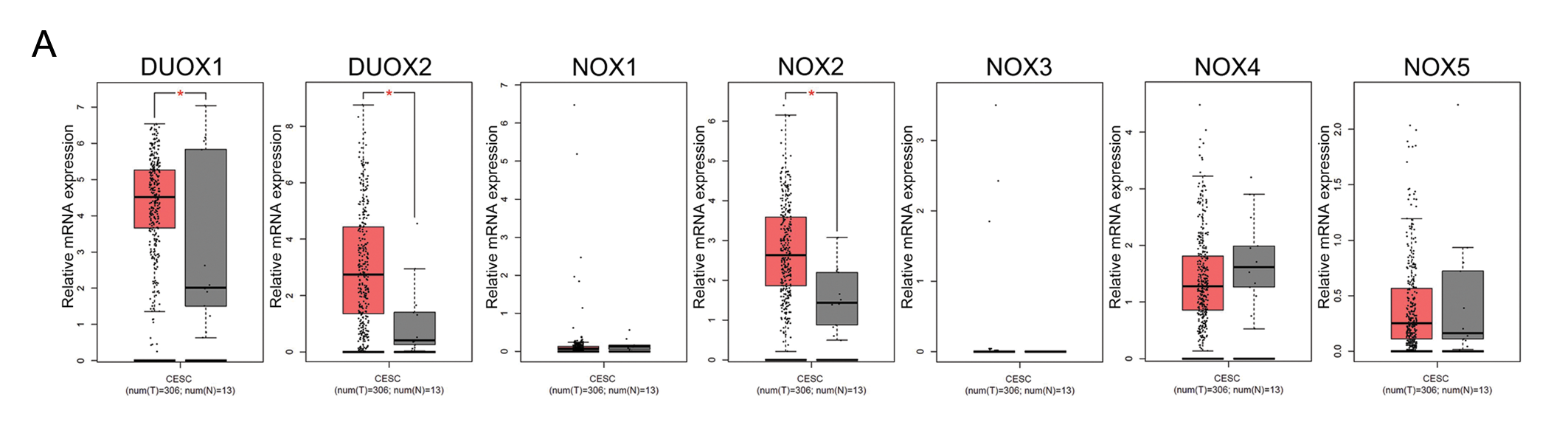

Supplement: Supplementary file 3 — Additional file 3. NOX family members expression in CESC, based on GEPIA database (Gene Expression Profiling Interactive Analysis). [file 12885_2019_6202_MOESM3_ESM.tif]

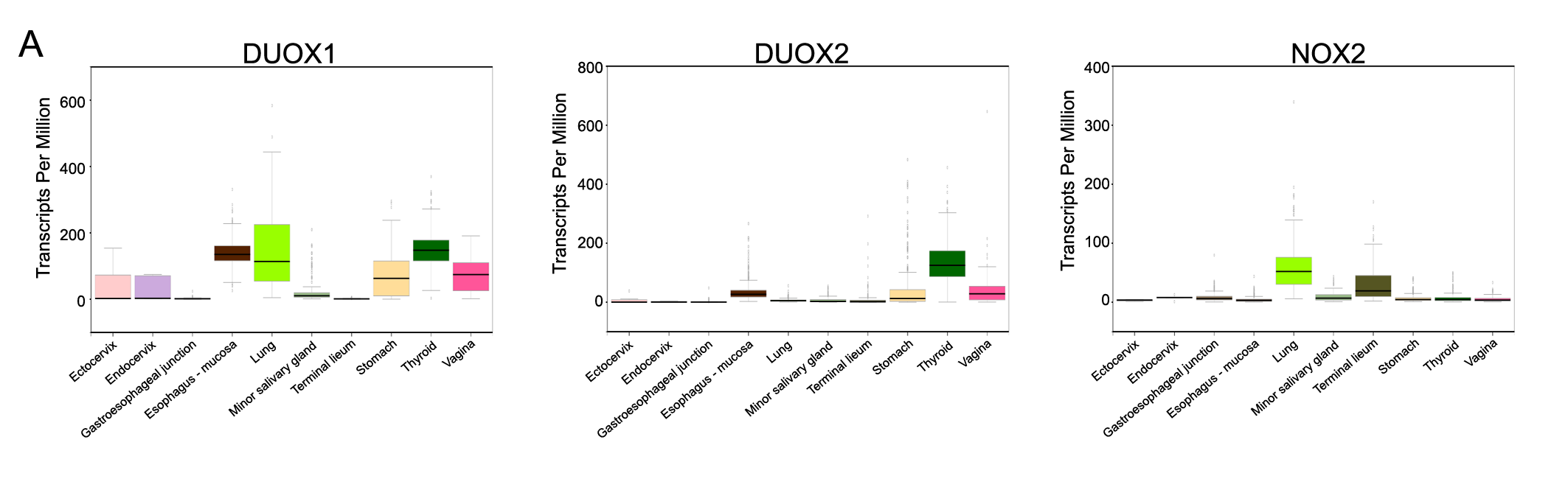

Supplement: Supplementary file 4 — Additional file 4. Tissue distribution of DUOX1, DUOX2, and NOX2 expression. RNAseq data were extracted from public data deposited by the Broad Institute of MIT and Harvard in the Gene Tissue Expression (GTEx) project. [file 12885_2019_6202_MOESM4_ESM.tif]

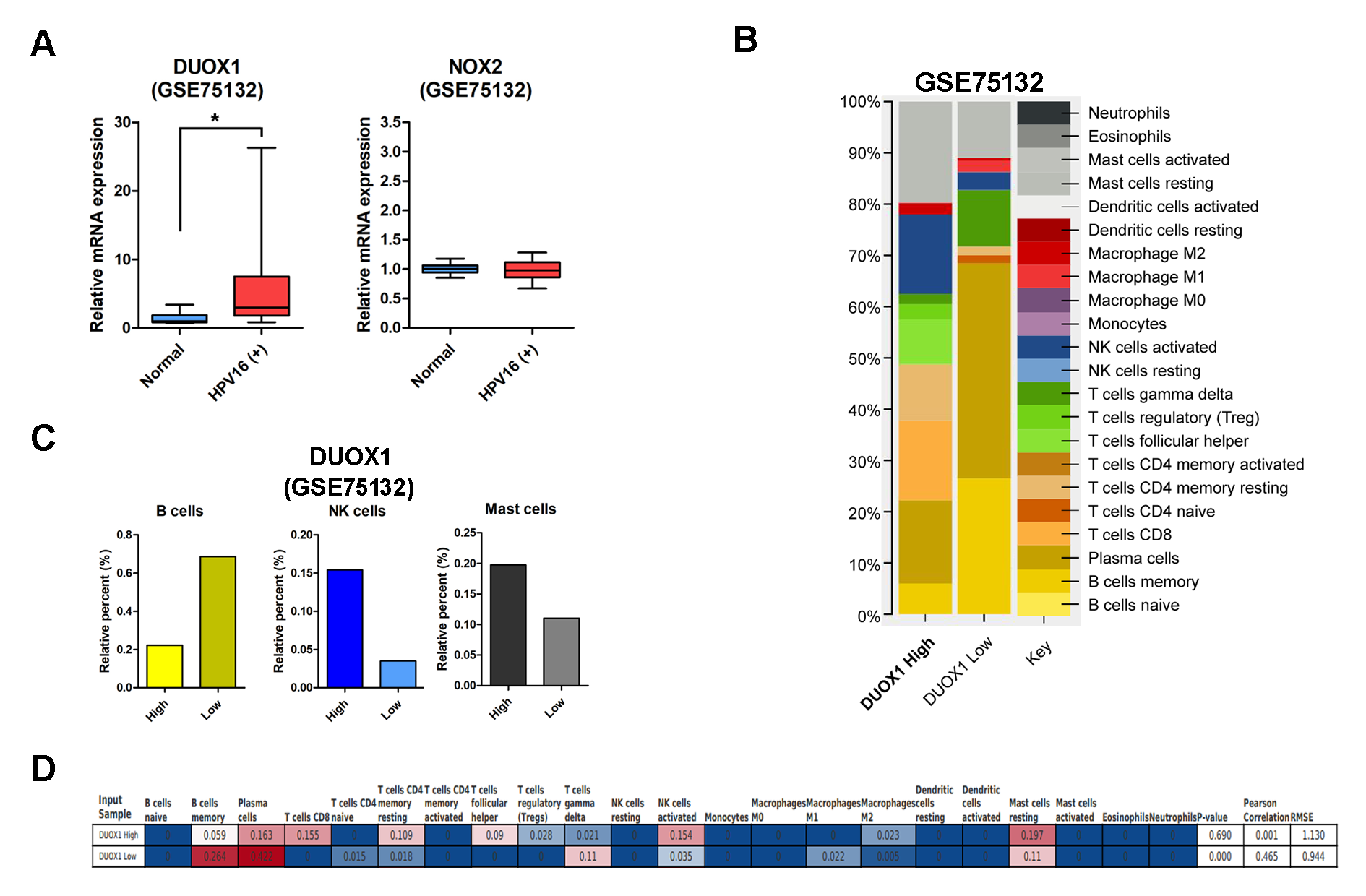

Supplement: Supplementary file 5 — Additional file 5. mRNA expression and Immune cell signatures in the validation data set (GSE75132). (A) mRNA expression of DUOX2 and NOX2 in patients with HPV 16 infection and normal control samples. (B) Relative percentages of LM 22 signature subsets in patients with DUOX1 gene expression. (C) Relative percentages of immune cells in patients with high and low DUOX1 gene expression. (D) Estimated percentage values of LM22 signature subsets, as calculated by CIBERSORT. [file 12885_2019_6202_MOESM5_ESM.tif]

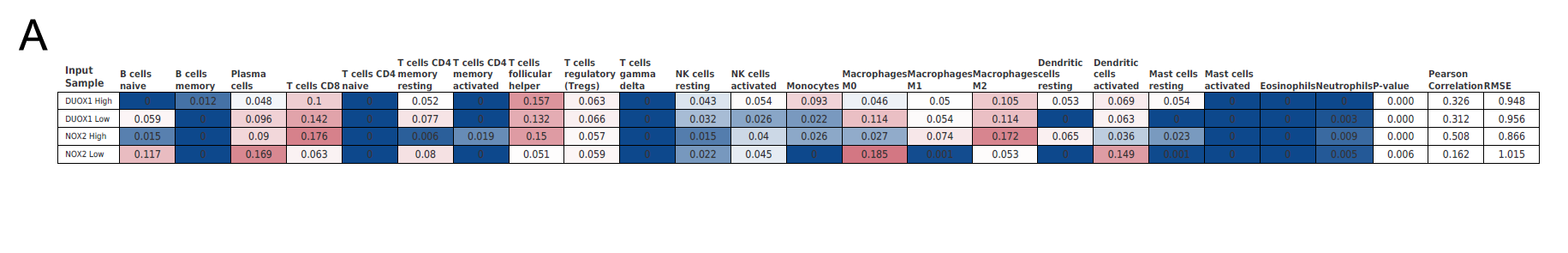

Supplement: Supplementary file 6 — Additional file 6. Estimated percentage values of 22 immune cell signature (LM22 signature) subsets, as calculated by CIBERSORT, between CESC patient groups in cervical cancer patients with DUOX1 and NOX2 gene expression. [file 12885_2019_6202_MOESM6_ESM.tif]
